# A golden decade for ocean science (2021–2030): from knowledge to solutions and actions

**DOI:** 10.1093/nsr/nwab021

**Published:** 2021-02-06

**Authors:** Weijie Zhao

**Affiliations:** NSR news editor based in Beijing

## Abstract

‘It is planning, it is science, it is management, and it is a huge change of human relations with the ocean.’ As introduced by Dr Vladimir Ryabinin, the Intergovernmental Oceanographic Commission (IOC) Executive Secretary and Assistant Director-General of the United Nations Educational, Scientific and Cultural Organization (UNESCO), the UN Decade of Ocean Science for Sustainable Development (the Decade, 2021–2030) got underway this January.

To echo that, on 14 January 2021, a Special Forum on the Decade was held in hybrid mode as part of the Fifth Xiamen Symposium on Marine Environmental Science (XMAS-V) in Xiamen University, China. The Forum was organized to promote the Decade through insightful talks and in-depth discussions with international and regional representatives who have been actively involved in the planning of the Decade. In addition to Dr Ryabinin, Zhanhai Zhang, Chief Engineer of the Ministry of Natural Resources of China, gave an inspiring opening speech. What followed were presentations by invited speakers. They then joined a panel discussion chaired by Dr Minhan Dai from Xiamen University. This article is edited and re-organized from the record of this Special Forum.

**Figure 1:**
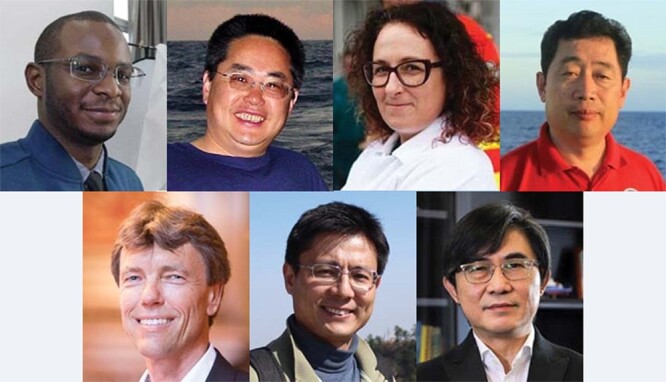
Panellists. First row from left to right: **Brandon Justin Bethel** (PhD Student of Marine Meteorology at Nanjing University of Information Science and Technology, China), **Fei Chai** (Professor at University of Maine, USA), **Karen Evans** (Principal Research Scientist of the Commonwealth Scientific and Industrial Research Organization (CSIRO) Oceans and Atmosphere, Australia), and **Fangli Qiao** (Senior Scientist at the First Institute of Oceanography, Ministry of Natural Resources, China). Second row from left to right: **Martin Visbeck** (Professor and Head of Research Unit, Physical Oceanography at GEOMAR Helmholtz Centre for Ocean Research Kiel, Germany), **Wenxi Zhu** (Head and Programme Specialist of IOC Sub-Commission for the Western Pacific (WESTPAC)), and **Minhan Dai** (Chair, Professor and Director of the State Key Laboratory of Marine Environmental Science, Xiamen University, China)

## ROADMAP OF THE DECADE: GLOBE, WESTERN PACIFIC AND CHINA

**Visbeck:** In the last several years, the United Nations proposed a number of multilateral processes to cope with global challenges, including the Paris Agreement, the New Urban Agenda and the 2030 Agenda for Sustainable Development. I had the opportunity to be part of the negotiation to set the 17 goals of sustainable development. I tried to make a very strong point that the ocean should receive an explicit goal, which turned out to be SDG14 (Sustainable Development Goal 14): Conserve and sustainably

The vision of the Decade is ‘The science we need for the ocean we want’.

—Martin Visbeck

use the oceans, seas and marine resources for sustainable development. Besides SDG14, the ocean is also mentioned in many of the other SDGs, such as No Poverty, Zero Hunger, Reduced Inequalities and Climate Action, etc.

On the other hand, the ocean science community wishes to contribute to these goals. They would like to learn more about the ocean, to support decision making, to safeguard the ocean and to help our economy to benefit from the ocean in a really sustainable way. There are many opportunities for ocean science and that is the policy backdrop of the Decade.

The vision of the Decade is ‘The science we need for the ocean we want’. What we are calling for is ‘transformative ocean science solutions for sustainable development, connecting people and our ocean’.

In order to do that, the executive planing group did a lot of consulting with scientists and stakeholders around the world, then came up with seven Decade Outcomes to describe the ocean we want: a clean, healthy and resilient, productive, predicted, safe, accessible, inspiring and engaging ocean.

For scientists, the Decade is proposing 10 Challenges that need to be addressed, including five Knowledge and Solutions Challenges, three Essential Infrastructure Challenges and two Foundational Challenges.

The Decade also set three technical Objectives and detailed Sub-Objectives. We are getting everybody—governments, academic communities, industries, private sectors, non-governmental organizations (NGOs), citizens and so on—together to take real actions.

The actions are organized in four different forms: programs, projects, activities and contributions. Actually, the first call for the Decade programs will be closed tomorrow (15 January 2021). It called for global and regional programs that could fulfill one or more of the Objectives. As an example, I am proposing a program on digital twins of the ocean.

Generally speaking, the Decade tries to transform the current ocean science and knowledge into real solutions and to transform the ocean we have into the ocean we want. It is a once-in-a-lifetime opportunity for everyone who cares about the ocean.

**Zhu:** Since the beginning of this millennium, the Western Pacific region has been a growth engine of the world economy and will lead economic recovery in the coming post-pandemic years. It is also one of the most populous regions, so human activity in this region has been inextricably linked to the ocean, triggering many grand challenges. For example, marine plastic pollution is extremely serious in this region.

Hence, achieving ocean sustainability in this region is essential for human's livelihoods and prosperities, not only for those living in the region but in the whole world. The UNESCO/IOC Sub-Commission for the Western Pacific (WESTPAC) considers the UN Decade of Ocean Science as a much needed opportunity to accelerate the development of ocean science solution to development challenges. As such it has been actively motivating countries and their institutions in the region to engage in, and contribute to the preparations of the Decade.

In line with the UN Ocean Decade, we organized the regional planning workshop in late 2019 and a Decade Regional Dialogue in late 2020 on `co-designing the ocean science we need for the ocean we want', building dialogues between various stakeholders for achieving the seven Decade Outcomes. We are also developing the UN Decade Regional Conference Series which will be held every three years in conjunction with the WESTPAC International Marine Science Conference and hosted by member states in this region on a rotational basis.

The Sub-Commission will step up its efforts in developing `solution-oriented' Decade programmes/projects. There are several in pipeline, including the programmes to combat marine plastic pollution and to develop an operational ocean forecasting system.

The WESTPAC has shaped its strategic direction for the Decade. We will encourage the engagement of early-career ocean professionals, advance the existing science-policy and science-innovation interfaces, develop and promote `co-designed, solution-oriented' ocean research programmes at local, national and international levels, leverage any potential financial support, and improve the ocean literacy of the public.

The Decade is a Decade for all stakeholders. We should think globally but act locally.

We should think globally but act locally.

—Wenxi Zhu

**Qiao:** Over the past 40 years, the economy has been growing rapidly in China, especially in the coastal areas. But it is accompanied by problems of marine pollution and unhealthy marine ecosystems. It is time for us to take actions to protect the ocean.

The Chinese government has proposed a series of strategies and policies that are consistent with the UN Ocean Decade and will actively take part in the Decade. We will soon establish a National Committee for the Decade and then coordinate all marine scientists and stakeholders to draft the National Action Plan for the Decade. After that, we will submit a proposal for the Decade. We are also planning to host an international Ocean Summit on the Decade in 2022.

For ocean science in China, we are facing several challenges, including insufficient observation and monitoring ability, inadequate understanding of global change and human impacts on the global ocean, and the science-based governance that we are not well equipped with.

The Decade provides us with a great opportunity to address these challenges. Many possible plans and actions for the Decade are emerging in China. For example, we would like to share our satellite technology and data with the global community to enhance the joint observation of the global ocean; we will share our knowledge and innovative theories to improve the prediction ability of the global ocean, typhoon and climate; we will also work with the WESTPAC to encourage the younger generation of ocean professionals from China, Asia and the globe.

I think the UN Decade is not just about ocean science, but also about the transformation of knowledge to solutions and actions. So, it needs action from all member states including China.

## SPIRIT OF THE DECADE

**Dai:** I would like to suggest three keywords for this panel discussion: challenge, engagement and action. Since we are getting down to implementing the Decade, I believe we should identify the challenges for the Decade, get partners from all sections engaged, and take action to transform ocean science and promote sustainable development in the next 10 years. As Martin introduced, the vision of the Decade is ‘The science we need for the ocean we want’. I would like to hear from you: how do you interpret this vision?

**Evans**: In my point of view, the Decade is a call for all of us to become involved in real actions to transform ocean science into solutions that can help us to obtain a truly sustainable future. We should think beyond the traditional scientific research mode.

**Chai:** The marine ecosystem has reached a critical point; many marine species are disappearing. We should arouse a sense of urgency in the global public. Humans have suffered a lot in the COVID-19 pandemic. However, in fact, the marine ecosystem and species have been suffering from pollution and destruction for a long time. But we, humans, did not see it.

**Bethel:** I was born in The Bahamas and have been studying in China for about 10 years. As a student, I have a lot of momentum to establish expertise and start my research career through the Decade. As a citizen of a small developing island state, I care about how the Decade will promote the development of a sustainable marine economy for small islands. I would like to join the Decade and make the ocean better for all.

I would like to suggest three keywords for this panel discussion: challenge, engagement and action.

—Minhan Dai

## GET MULTI-STAKEHOLDERS ENGAGED

**Qiao:** I think a fundamental question for the Decade’s actions is who the main actor should be. To my understanding, firstly, state governments should play a key role, because we need strong financial support and all kinds of resources for the actions. Secondly, the UN agencies, especially the local international organizations, such as the WESTPAC, should also play a key role, because they know much about the challenges for each state in the area so they can better lead the way. And of course, the scientific community should be a major actor. Scientists should

The structure of the Decade programs is different from traditional science programs. It requires participants that are not only scientists but also other stakeholders.

—Karen Evans

provide solutions instead of just publishing research articles. Also, they should better communicate with the public and encourage the whole society to take action.

**Dai:** Personally, I have not seen much engagement in the industry. Martin, would you please introduce the related situations and plans?

**Visbeck:** The Decade is just beginning, and we are still thinking about how to best implement it. I am advocating for a special Private Sector Panel to promote the engagement of the business sector.

Actually, we have quite a few private sector actors already engaged in the Decade. For example, there is a company called Fugro that offers consultations and services related to energy, environment and also mariculture installations. Another example is Esri, which is a geographic information system (GIS) provider for the Decade. There are also instrument providers. Companies, such as Google, are also willing to offer services to the Decade.

I think we will see a lot more companies involved in the next year when it becomes obvious to them how they can be engaged in, and benefit from, the Decade programs.

One of the reasons why we do not hear so much from them is that the way in which we debate about the Decade, like the Forum today, is not so interesting for the private sector actors. They prefer faster-paced meetings focusing on certain business problems and models. So, in our future discussions, we should consider how to attract their attention.

**Dai:** That is encouraging. The next question is, how will the Decade help build science-policy and science-industry interfaces, and encourage solution-oriented research?

**Evans:** In developing the Decade, it is really important to make connections between groups that perhaps had not worked closely together in the past. Actually, the structure of the Decade programs is different from traditional science programs. It requires participants that are not only scientists but also other stakeholders. We have designed mechanisms to make connections between those who have been completely engaged in science, who straddle science, management and policy, just like myself, as well as those who are actually making decisions.

**Visbeck:** I would like to provide an analogy. In the current coronavirus pandemic, we can see that in a hospital there are different types of health workers. Some take care of the patients, some analyze the viral sequence and some others work with the vaccines. Their work is different, but they work as a team. I hope that in 2030, ocean science will be the equivalent of the hospital; that scientists, inventors, industries, policymakers and the public will work as a team. I think during the Decade, we have to think about how to learn from medical science to promote multi-stakeholder engagement.

**Audience-1**: Besides scientists and engineers, a group of people are technology innovators. It is sometimes hard to classify them as scientists or engineers. They can be both.

**Chai:** The most dangerous thing is to put a label on an individual. We need all kinds of problem solvers to solve complicated ocean issues. There is no single category of people, scientists or engineers, that can solve the problems all by themselves. I like Martin's hospital analogy. We need to build a modern hospital of ocean science with multiple problem solvers.

## EMPOWER THE YOUNGER GENERATION

**Chai:** I think another major challenge of the Decade is the training of the future generation of scientific leaders. Again, it is a once-in-a-lifetime opportunity for young people. If they participate in and contribute to the programs, at the end of the Decade they will become leaders of ocean science.

The future belongs to young generations. They cannot rely on previous generations to give them the ocean they want. So, they should have a sense of responsibility and motivate themselves for action. For instance, I was impressed by the videos played at the beginning of the Forum, which were made by the young student team here. This is the beginning of their involvement.

**Zhu:** This is a competitive world with limited resources and opportunities. Thus, our early-career ocean scientists must be proactive and self-prepared to get the opportunities. The WESTPAC has plans to help early-career scientists, including supporting them to attend regional conferences and providing them with training opportunities.

**Evans:** From the very beginning, we designed an early-career ocean professional network for the Decade, which provides opportunities for all ocean professionals to participate in the Decade. We want as many early-career ocean professionals as possible to take the opportunity and get involved in the network.

**Dai:** Since we are talking about the younger generation, Brandon, would you like to share your views? Do you have experiences of similar programs, and what are the potential difficulties for young scientists?

**Bethel:** I have taken part in a program in 2015 in Zhejiang Province, China. During the program, we worked on various aspects of the ocean. We woke up at six o’clock and worked long hours until the work was done. While I did not experience any difficulty, I do have something to share. I am now a physical oceanographer, and my work concerns waves and ocean surface winds. But during the program, I worked with biological oceanographers, who worked with ocean animals such as fish and shrimp, which are much more relatable to newcomers to the field. So, I think many young people in my position can get involved in programs like that.

**Dai:** Thank you for sharing. As senior scientists and also educators, how can we motivate young students to be major contributors or leaders?

If they participate in and contribute to the programs, at the end of the Decade they will become leaders of ocean science.

—Fei Chai

**Chai:** I think we should promote cross-disciplinary education. The old way is to train individual students to be specialized scientists such as physical oceanographers or chemical oceanographers. But these disciplines speak different vocabularies and think in different ways. So, it is difficult for them to cooperate. In the training of the younger generation, we should train them in cross-disciplines from the very beginning. We can encourage them to get a second degree or transfer to a different major in the Master or PhD stage. We can also encourage them to study in different countries so that they will feel free to cooperate globally.

**Dai:** Thank you. It is very important to adjust the mode of current ocean science education in universities. I think the UN Ocean Decade sets a very good example of how to mobilize young scientists not only doing their own science but also thinking about real society, thinking about solutions for a better future.

**Audience-2:** How can young students get connected with the government system?

**Visbeck:** In Germany and the US, there are intern opportunities for Master or PhD students to spend a year or two in the political system. They can learn the language of politicians, and the politicians also like that because they need scientific information. I think it is a good idea to encourage such interns in political systems and also private sectors. The Decade will also provide that kind of opportunity for young students.

## IMPROVE OCEAN LITERACY OF THE PUBLIC

**Bethel:** I am from the Caribbean. Where I live, most people are in very close contact with the ocean every day, but they do not see things beyond blue color of the ocean. They do not see it as the source of energy or the key to combating climate change. When I try to explain my work as a physical oceanographer, they are often confused as to what I am talking about. Therefore, it is an urgent task to improve the ocean literacy of the public.

**Dai:** How can we do that efficiently?

**Visbeck:** My answer is very simple. Most of us humans enjoy stories, so we can tell interesting stories about the ocean. For example, we can talk about how ocean scientists actually work on ships. Also, I know that in China, a lot of people want to take wedding photos on the coast and many people take trips to the southern coast in the winter months. These are all good points to develop and tell stories about ocean pollution and ocean science.

Another powerful way to tell stories is through television and movies. For example, the BBC films about biodiversity and the ocean got enormous attention in the English-speaking world. When they are translated to German, Chinese or other languages, they will also be powerful stories for the public.

Today, games and social media are also influential. We can work with those communities, for instance, work with the media stars, to tell stories about the ocean and reach out to much more people.

Of course, this is not a job for just scientists. The governments and the NGOs should get involved to provide resources and take action.

**Audience-3:** To improve public ocean literacy, is it a good idea to start with the kids and the teenagers?

**Bethel:** Yes, and if you want to get to the kids and teenagers, it is a great idea to go to the movies. Movies can present the ocean in such a way that even those who were not knowledgeable of the ocean can understand and enjoy it. For example, we all know Disney movies such as *TheLittle Mermaid* and *Finding Nemo*, which are really great ocean stories for kids.

Where I live, most people are in very close contact with the ocean every day, but they do not see things beyond blue color of the ocean.

—Brandon Justin Bethel

## CHALLENGES THROUGHOUT THE WORLD

### China and Asia

**Dai:** What do you think are the major challenges for China, with regard to participating in the Decade?

**Visbeck:** It is extremely impressive to hear about China's proactive engagement in the Decade. I see two challenges for China. First, how can China maximize its action in cooperating with other countries and training young scientists from other parts of the world? Second, under Chinese culture, how can we get broad society engaged? I think you can take advantage of the visibility of the Decade to really reach out to society.

**Zhu:** For China, and many other countries, the language barrier is a great challenge. Since the working language of most international programs is English or French, it is difficult for Chinese people, especially the broad public, to get informed and engaged. Considering the large world populations that do not take English as their mother tongue, the language barrier is a great challenge for the future of ocean science.

**Qiao:** I think for China, the biggest challenge is to attract more attention and support from the government. All the Decade actions need financial, policy and organizational support from the government. It is the key to success. I hope that we can promote more communication between ocean scientists and different government agencies, including the Ministry of Natural Resources and the Ministry of Science and Technology.

Secondly, the science community of China, including the young students, is actively engaged in the Decade. But that is

For China, the biggest challenge is to attract more attention and support from the government.

—Fangli Qiao

not enough. We need to attract the attention of the public and different stakeholders.

**Dai:** Thank you. Let us turn from China to Asia. What are the challenges we are facing in Asia in terms of promoting the Decade?

**Zhu:** Asia is a very important area for the global ocean—its ocean area is huge, it is populous, and it has the highest marine biodiversity worldwide. Without a clean and healthy ocean in Asia, we will not get a sustainable ocean for the world.

The WESTPAC is doing everything possible to build partnerships and promote the implementation of actions in this region. But we have to admit that within the UN system, the voice from this region is very limited. I think within the whole UN system, the number of ocean professionals from this region is less than five. So, there are still many challenges facing this region, especially for the developing areas.

### Inland areas

**Audience-4:** My hometown is in inland China. How can we inform the inland people of the importance of the ocean, and how can they contribute to the Decade?

**Chai:** The ocean is a great contributor to the prosperity of inland areas. For example, today we can see Japanese restaurants in most cities throughout the world, where you can enjoy fresh seafood from different parts of the world. Without a healthy and clean ocean, we will not be able to enjoy that. Moreover, a safe ocean is essential for global trading, without which the inland citizens may not be able to buy clothes, toys, cellphones or cars made on the other side of the world. So, you can tell these stories to your friends and families and they will understand and care about the ocean.

**Visbeck:** We have talked about how to improve the ocean literacy of the public, and I think that works also for the inland public. And, even if you live in inland areas, you can also do something for the ocean. For example, you can refuse to buy products made of shark fins. All these behaviors are helpful for a sustainable ocean.

### Small islands and developing states

**Dai:** In the Decade, what would be the role of governments and citizens in small islands and developing states?

**Evans:** In the South Pacific and Western Central Pacific areas, the governments of small islands and developing states are organized by the Pacific Community, which is an organization that conducts science on behalf of those countries. The Pacific Community will form a Center for Ocean Science in the Pacific, which will be the major force to bring together these Pacific islands for the Decade.

In Western Central Pacific, a lot of marine resources are actually managed by local community groups. These community groups are actively engaged in the Decade. Actually, a lot of the Decade initiatives concerning ocean reserves and ocean management were from the communities, rather than the governments. The governments are interconnectors, but the communities are the real driving forces.

**Zhu:** Small islands and developing countries are very active in the Decade. Their voices are well heard not only at the IOC level but also at the UN level. Other communities and countries are also starting to recognize the importance of small islands and some companies are willing to offer services for these pacific islands. Moreover, early-career ocean professionals from these areas are actively engaged in the Decade. They are setting a good example for global young ocean professionals.

**Dai:** Thank you all for joining this Special Forum and sharing your information and insightful views about the Decade. I hope that in the coming 10 years, we can fully take the opportunity of the Decade and contribute our efforts to transforming ocean science and generating more solutions for a sustainable future ocean.

